# The Endocrine System: Alcohol Alters Critical Hormonal Balance

**Published:** 1997

**Authors:** Nicholas Emanuele, Mary Ann Emanuele

**Affiliations:** Nicholas Emanuele, M.D., is professor of medicine and director of the Division of Endocrinology and Metabolism, Loyola University Medical School, Maywood, Illinois, and Hines VA Hospital. He is also a member of the Division on Research on Drugs of Abuse. Mary Ann Emanuele, M.D., is professor of medicine, Division of Endocrinology and Metabolism, Loyola University Medical School, Maywood, Illinois, and Hines VA Hospital. She is also professor of molecular and cellular biochemistry and a member of the Division on Research on Drugs of Abuse

**Keywords:** AODE (alcohol and other drug effects), AODR (alcohol and other drug related) disorder, endocrine function, endocrine disorder, gonad function, hormones, cardiovascular disorder, immune disorder, bone, calcium, pancreas, drug therapy, literature review

## Abstract

Alcohol’s effects on the hormonal (i.e., endocrine) system have widespread consequences for virtually the entire body. Alcohol-related hormonal disturbances can result in cardiovascular abnormalities and reproductive deficits in both males and females. Other endocrine problems stemming from excess alcohol consumption include immune dysfunction and bone disease. Researchers are exploring ways of using hormonal mechanisms to help treat alcoholics as well as to identify people predisposed to alcoholism.

Along with the nervous system, the endocrine, or hormonal, system is the primary regulatory mechanism for virtually the entire human body. Hormones are chemical messengers that control and coordinate the function of tissues and organs. Each hormone is secreted from a particular gland and distributed throughout the body to act on different tissues.

Hormones are released as a result of nerve impulses or in response to specific physiological or biochemical events. Following their release, hormones instigate a cascade of reactions within the body, the end result of which can include synthesis and release of enzymes and changes in cell membranes. Highly sensitive feedback mechanisms reduce or increase the amount of different hormones being released at any given time.

The effects of alcohol on endocrine function are multiple and complex. Several variables, including the type, length, and pattern of alcohol exposure; level of intoxication; and coexisting medical problems, such as malnutrition and liver dysfunction, must be considered when assessing the impact of alcohol on hormonal status. This article summarizes the effects of both acute and chronic alcohol exposure on hormonal physiology, addressing work performed on humans and animal models.

## Hypothalamic-Pituitary-Adrenal Axis

The hypothalamus is the control center for most of the body’s hormonal systems. Located deep within the brain, the hypothalamus receives nerve impulses stemming from both physical and psychological stimuli and releases hormones in response to those signals. Hypothalamic activity thus governs numerous body functions, including reproduction, metabolism, use of nutrients, and growth.

**Figure 1:**
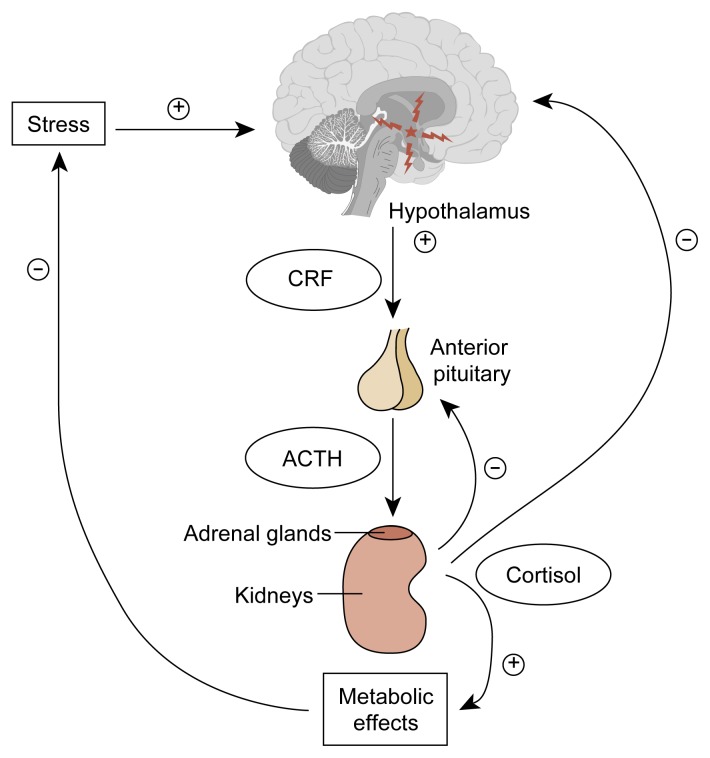
The hypothalamic-pituitary-adrenal axis. In response to almost any type of stress, either physical or psychological, the hypothalamus secretes corticotropin-releasing factor (CRF), which in turn increases secretion of adrenocortotropic hormone (ACTH) by the anterior pituitary gland. In response, within minutes, the adrenal glands, located atop the two kidneys, increase secretion of cortisol. The released cortisol initiates a series of metabolic effects aimed at alleviating the harmful effects of the stress state and, through direct negative feedback to both the hypothalamus and the anterior pituitary, decreases the concentration of ACTH and cortisol in the blood once the state of stress abates. ⊕ = excites ⊝ = inhibits

The hypothalamus, the anterior pituitary gland, and the adrenal glands function together as a well-coordinated unit known as the hypothalamic-pituitary-adrenal (HPA) axis (see figure 1). Cells in the hypothalamus (i.e., the paraventricular nucleus) produce most of a key hormone called corticotropin-releasing factor (CRF) in humans. The hypothalamus secretes CRF into the hypothalamic-pituitary portal system, the network of blood vessels that functionally connects the hypothalamus and the anterior pituitary gland. At the pituitary gland, CRF binds to specific receptors on special pituitary cells called corticotropes, which produce adrenocorticotropic hormone (ACTH). Upon CRF stimulation, ACTH production and secretion is enhanced. ACTH is then transported through the general blood circulation to its target tissue, the adrenal gland, where it stimulates production of adrenal hormones, primarily glucocorticoids.^1^ Glucocorticoids then feed back in a negative fashion^2^ to both the hypothalamus and the pituitary gland to decrease CRF and ACTH release, respectively (Miller and Tyrrell 1995).

Glucocorticoids have many physiological effects; they influence carbohydrate, lipid, protein, and nucleic acid metabolism; the cardiovascular system; bone and calcium metabolism; the central nervous system; and growth, development, and reproduction. Notably, glucocorticoids also modify immunological responses, underscoring the important interrelationship between the endocrine and immune systems. In addition to their functions within the HPA, both CRF and ACTH have independent effects on the immune system, reproduction, and temperature regulation (Miller and Tyrrell 1995). Although increases in ACTH and glucocorticoids are usually short-lived (i.e., transient), this stimulation has far-reaching consequences for numerous other organ systems. It is easy to appreciate how perturbations of the system may lead to multiple clinically significant problems.

### Animal Studies

The major circulating glucocorticoid in humans is cortisol; in rodents, it is corticosterone. In rats, it is clear that acute alcohol administration leads to dose-related increases in ACTH and corticosterone, with females showing a greater response than males.^3^ Consistent evidence shows that alcohol’s effect is through enhanced release of CRF. For example, destruction of the paraventricular nucleus (the hypothalamic area where CRF is made), neutralization of naturally occurring CRF with antibodies, or blockade of CRF receptors with certain chemicals all interrupt the ability of alcohol to stimulate the HPA. Furthermore, incubation of rodent hypothalamic slices in alcohol leads to acute release of CRF (Pascal de Waele and Gianoulakis 1993). Although there is no question that acute alcohol administration stimulates CRF release, it remains unresolved whether this stimulation occurs through a direct effect on CRF-producing neurons in the hypothalamus or indirectly, through other input from the hypothalamus or other areas of the brain (i.e., extrahypothalamic input) to CRF neurons.

In addition to having effects on the brain (whether hypothalamic, extrahypothalamic, or both), it is conceivable that alcohol might stimulate ACTH or corticosterone release, acting directly at the pituitary gland or at the target organ (i.e., the adrenal cortex). However, no consistent pattern of response has been observed. Some studies suggest a direct pituitary stimulation of ACTH release, yet others show no direct effect of alcohol. Similarly, some data show a direct stimulatory effect of alcohol on adrenal cortex, whereas other findings show no change.

Thus, in animals, *acute* alcohol administration transiently activates the HPA axis, mainly by stimulating release of hypothalamic CRF, which then enhances ACTH and subsequent corticosterone release. The HPA axis of rodents *chronically* exposed to alcohol, however, remains activated (i.e., ACTH and corticosterone levels are increased compared with levels in control animals), although some degree of tolerance develops. That is, the HPA axis is more activated after chronic alcohol exposure than after no exposure, but not as activated as after acute alcohol exposure. As is the case with acute alcohol administration, chronic alcohol exposure seems to cause hyperactivity of the HPA axis through effects, either direct or indirect, on CRF. Levels of CRF messenger RNA (mRNA) are an indicator of the rate of CRF synthesis and overall CRF levels. Compared with control animals, chronically alcohol-exposed animals show increased baseline (i.e., basal) levels of CRF mRNA and decreased hypothalamic CRF content. These data suggest that chronic alcohol exposure increases the synthesis and release of CRF, a theory that has been confirmed in a study of rats by Redei and colleagues (1988). The increased release of CRF would explain the increased levels of ACTH and adrenal corticosterone. As indicated above, because of tolerance the level of HPA activation after chronic alcohol exposure is not as great as following acute alcohol exposure.

What mechanism could account for this tolerance? First, at the brain level, in hypothalami from rats chronically exposed to alcohol, CRF release is not as high as in control, non-alcohol-exposed animals. Second, at the pituitary level, chronic alcohol exposure decreases both pituitary CRF binding and the activity of adenylate cyclase, a enzyme key to normal cell function. The decreased binding of CRF to the corticotropes at the pituitary gland may be a direct effect of alcohol or may result from the alcohol-induced increase in CRF release; like many hormones, CRF down-regulates its binding to receptors as a result of feedback from the HPA axis. In any case, compared with the pituitary glands of control rats, pituitaries of rats chronically exposed to alcohol demonstrate decreased ACTH output. Thus, in rats chronically treated with alcohol, a given amount of CRF produces less of an ACTH response and consequent diminished corticosterone output than in control rats; in other words, tolerance develops. The apparent reason that the HPA axis is still activated after chronic alcohol administration is that CRF secretion remains, overall, above control levels.

Interestingly, age may modify the ability of chronic alcohol administration to activate the HPA. Although in young rats (i.e., ages 3 to 6 months), alcohol can chronically stimulate HPA activity, alcohol actually decreases ACTH and corticosterone activity in “middle-aged” (i.e., 9-month-old) rats. Chronic alcohol adminstration causes a profound decrease in HPA activity in “elderly” (i.e., 15-month-old) rats (Nolan et al. 1991).

As previously indicated, the functions of the endocrine and immune systems are intimately interconnected. For example, alcohol-exposed rats have an impaired ACTH response to interleukin-1α and interleukin-1β, two chemicals key to the overall immune response that are produced by immune-system cells. Alcohol-induced dysfunction (i.e., hyperactivity) of the HPA axis may alter immune function and thus the capacity of the body to stave off infection, an ability compromised in alcoholic humans. (For more information on alcohol’s effects on the immune system, see the article by Szabo, pp. 30–41.)

### Human Studies

One manifestation of alcohol’s effect on the HPA axis resembles a disorder called Cushing’s syndrome, a disease stemming from an excess of cortisol. Clinical signs of the syndrome include obesity of the torso (with purplish stretch marks); a round, red face; high blood pressure; muscle weakness; easy bruisability; acne; diabetes; osteoporosis; and a variety of psychological disturbances. Women also may develop facial hair (i.e., hirsutism) and menstrual disturbances. Some instances of Cushing’s syndrome are caused by prolonged use of high-dose glucocorticoids as therapy for other medical conditions; aside from drug-induced Cushing’s syndrome, however, about two-thirds of the cases of Cushing’s syndrome are caused by ACTH-producing pituitary tumors. The therapy for the syndrome is surgery to remove the tumors. Another 15 percent of Cushing’s cases result from an adrenal tumor, and the remainder of cases are generally caused by nonpituitary and nonadrenal tumors (generally seen in the lung, the pancreas, and the gut) that abnormally produce ACTH. Therapy is the surgical or, in some cases, pharmacological treatment of these tumors.

Some drinkers develop a condition called alcohol-induced pseudo-Cushing’s syndrome. It is indistinguishable from true Cushing’s syndrome, although it tends to be clinically more mild. Proof that the pseudo-Cushing’s syndrome results from alcohol consumption and not from tumorous overproduction of ACTH or cortisol derives from the observation that its symptoms and signs disappear with abstinence from alcohol, usually within 2 to 4 months. The prevalence of this syndrome among alcoholics is unknown, but clinical experience indicates that most alcoholics do not have the full-blown syndrome.

Based on alcohol’s ability to activate the HPA axis in animals (Thiagarajan et al. 1989), the existence of alcohol-induced pseudo-Cushing’s syndrome should not be surprising. Whether the syndrome results from alcohol’s effects on the brain or at the pituitary or adrenal levels, however, is not clear. Nonetheless, the existence of alcohol-induced pseudo-Cushing’s syndrome indicates that alcohol consumption somehow leads to a clinically significant activation of the HPA axis in humans. (For a recent review of the data on alcohol’s effects on the HPA axis in alcoholics and nonalcoholics, see Veldman and Meinders 1996.)

## Hypothalamic-Pituitary-Gonadal Axis

**Figure 2:**
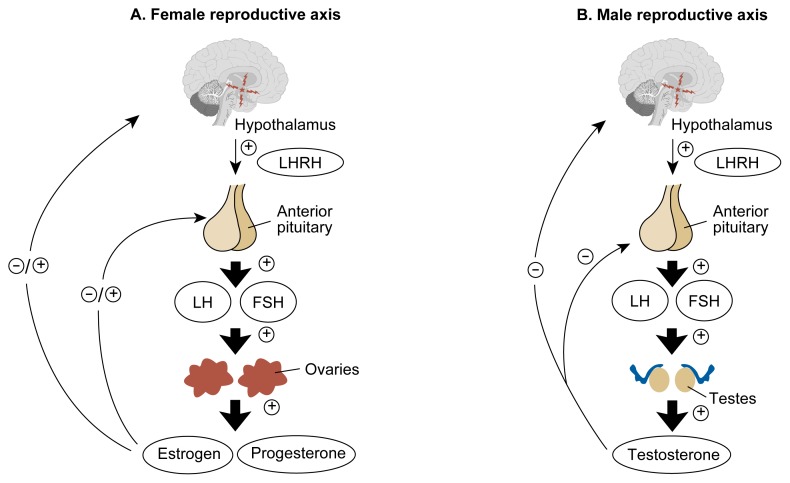
The hypothalamic-pituitary-gonadal axis in males and females. The hypothalamus secretes luteinizing hormone-releasing hormone (LHRH), which controls the anterior pituitary gland’s secretion of both follicle-stimulating hormone (FSH) and luteinizing hormone (LH). In the female reproductive axis (A), these two gonadotropic hormones stimulate the ovaries to secrete estrogen and progesterone, which circulate back to the hypothalamic-pituitary unit and either inhibit or excite the production of FSH, LH, and LHRH. In the male reproductive axis (B), the gonadotropic hormones stimulate production of testosterone by the testes; testosterone also feeds back to the hypothalamus and pituitary to inhibit production of LHRH and the pituitary gonadotropins. ⊕ = excites ⊝ = inhibits

The hypothalamic-pituitary-gonadal axis is a complex system involving feedback from the target organs, the gonads (i.e., the testes and ovaries), to the hypothalamus, where a key reproductive hormone, luteinizing hormone-releasing hormone (LHRH), is released into the portal blood system (figure 2). Upon reaching the pituitary gland, LHRH attaches to specific receptors and activates a complicated cascade of biochemical events that results in the synthesis and release of the two gonadotropin hormones, luteinizing hormone (LH) and follicle-stimulating hormone (FSH).

LH is largely responsible for gonadal production of androgens, which are hormones that have masculinizing effects (e.g., testosterone). FSH is important for normal development and maturation of sperm in the male and ovarian follicles in the female. The gonadal hormones—including testosterone in the male and estrogen and progesterone in the female—then circulate back to the hypothalamic-pituitary unit and encourage or discourage further release of LHRH, LH, and FSH in a finely tuned system (Molitch 1995; Santen 1995; Erickson 1995).

**Figure 3:**
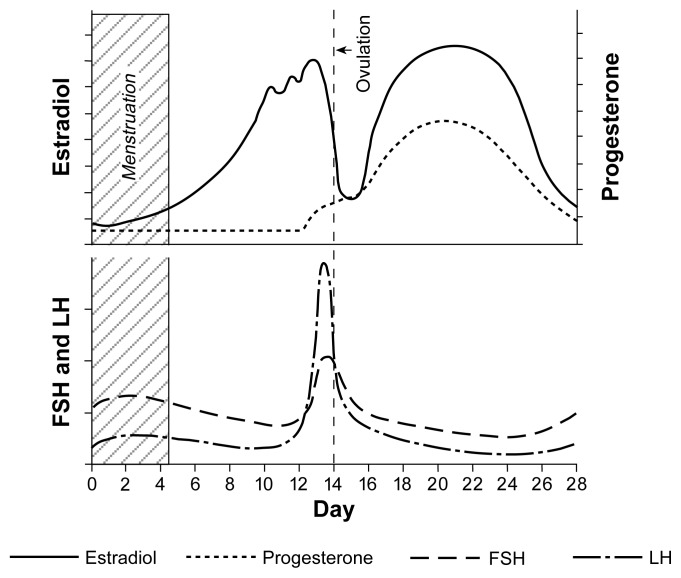
Fluctuations in the levels of estradiol, progesterone, luteinizing hormone (LH), and follicle-stimulating hormone (FSH) over the 28-day female reproductive cycle. SOURCE: Adapted from Guyton, A.C. *Textbook of Medical Physiology*. 7th ed. Philadelphia: W.B. Saunders, 1986. p. 969.

Because of female reproductive cyclicity, the hormonal physiology is more complex in women than in men (Erickson 1995). The 28-day human reproductive cycle can be divided into two phases: a 14-day follicular phase, which begins with the first day of menses, followed by a 14-day luteal phase. Ovulation, the release of the egg (i.e., ovum) from the ovary, occurs at the midpoint of the 28-day cycle (see figure 3).

The ovary is made up of millions of follicles, each consisting of a single ovum in a multilayered envelope of hormone-producing cells called granulosa cells. During each reproductive cycle, just one of the follicles is recruited to become the dominant follicle, which will ovulate. (The process by which only one follicle per cycle becomes dominant is not well understood.) FSH is the key pituitary hormone of the follicular phase; under FSH stimulation, the dominant follicle undergoes a normal developmental process. As the follicular phase progresses and the ovary is readied for ovulation, the granulosa cells multiply and secrete increasing amounts of the estrogen called estradiol.

Estradiol causes increased thickness of the uterine wall, preparing it for implantation of the fertilized ovum. Estradiol also feeds back to the hypothalamic-pituitary unit to cause two important effects: First, through negative feedback, estradiol progressively decreases output of FSH. Second, estradiol sensitizes the pituitary gland to the effects of LHRH. The second function is important because at the end of the 14-day follicular phase (i.e., at midcycle), progressively rising estradiol emanating from the dominant ovarian follicle causes a burst of LHRH secretion from the hypothalamus into the portal blood system, causing a large midcycle surge of LH and FSH secretion, which in turn triggers ovulation and the start of the luteal phase.

Following ovulation, the granulosa cells of the dominant follicle undergo a process called luteinization, converting the follicle into a body called the corpus luteum. The corpus luteum is the key endocrine structure of the luteal phase; its major secretory product is the hormone progesterone, which further prepares the uterine wall for a fertilized ovum and is crucial to the successful maintenance of early pregnancy.

Although the products of the gonads are essential to reproduction, testosterone, estrogen, and progesterone, like the glucocorticoids, all have actions throughout the body. These effects include roles in carbohydrate and lipid metabolism, the cardiovascular system, and normal bone growth and development. In addition, gonadal hormones are important mediators of the central nervous system and play a role in the immune system. A disturbance of the hypothalamic-pituitary-gonadal axis thus can result not only in altered fertility but also in problems such as osteoporosis, muscle weakness, and impaired immune function.

### Animal Studies

The effects of both acute and chronic alcohol administration have been studied primarily in male rats. (Although studies in females exist, they are relatively few in number, and thus the data on males will be presented first.) A direct effect of alcohol on testosterone secretion in male rats has been amply documented (Cicero 1982; Emanuele et al. 1993; Purohit 1993), and a secondary impact of alcohol on the hypothalamic-pituitary unit also has been noted. Animal studies measuring concentration of LHRH in the hypothalamic-pituitary portal system have found a sharp fall in LHRH secretion after an acute alcohol injection in anesthetized male rats (Ching et al. 1988). Studies on rat hypothalami, however, have failed to detect an alteration in either basal or stimulated LHRH release after exposure to alcohol (Emanuele et al. 1990). Thus, the hypothalamic effects on LHRH may be mediated in brain areas outside the hypothalamus, indirectly affecting hypothalamic function. The result is a reduction in (i.e., attenuated) LHRH release and, consequently, a diminished gonadotropin response.

Researchers studying the effects of alcohol on the mRNA of LH, an indicator of the rate of LH synthesis, have observed a drop specifically in pituitary LH RNA levels in rats (Emanuele et al. 1993). The lowered amount of LH being produced by the pituitary results in lowered testosterone levels because the gonads are unstimulated. In addition, alcohol directly affects the Leydig cells, the testosterone-producing cells of the testicles, which demonstrate marked inhibition of testosterone secretion after acute alcohol exposure. Thus, acute alcohol administration to adult male rodents results in lowered testosterone levels through both a central effect on the hypothalamic-pituitary unit and direct suppression of Leydig cell activity.

Over time, the hypothalamic-pituitary unit appears to become somewhat resistant to the deleterious consequences of alcohol, although testosterone levels remain low. It has been noted that the hypothalamic-pituitary unit of male rodents chronically exposed to alcohol demonstrated little or no change in hypothalamic LHRH and the rate of pituitary LH and FSH production (Emanuele et al. 1993). In theory, hypothalamic LHRH and pituitary LH and FSH levels should actually increase in an attempt to stimulate the gonads to produce testosterone. Thus, the lack of change in LHRH, LH, and FSH levels after chronic alcohol exposure is inconsistent with the presence of a low testosterone level, indicating that the hypothalamic-pituitary-gonadal axis is relatively suppressed by alcohol.

### Studies in Men

Diminished sexual function in alcoholic men has been clinically noted over many years; early studies largely attributed this problem to liver disease. However, when young, healthy, non-alcoholic volunteers were exposed acutely to alcohol, a fall in serum testosterone was consistently demonstrated. A central defect in the human hypothalamic-pituitary unit was first suggested when it was noted that LH levels in adult men failed to increase as expected for the concomitant decrease in testosterone levels, and both LH and FSH failed to increase after stimulation with clomiphene citrate, a drug that acts at the hypothalamus level to increase LHRH production (Cicero 1982). Interestingly, in younger men who were given alcohol and LHRH to stimulate their pituitary glands’ production of LH, testosterone levels actually increased, suggesting enhanced sensitivity. Thus, a central effect of alcohol in humans also has been implied after acute alcohol exposure.

### Studies in Women

Even moderate drinking in healthy women can lead to significant reproductive problems. (For a review, see Mello et al. 1993). Mendelson and Mello (1988) studied a group of healthy, well-nourished women during a 35-day stay on a clinical research unit. The first and last 7 days were alcohol-free. During the middle 21 days, each participant could drink alcohol as desired. At the end of the 35 days, the women classified themselves as heavy drinkers (7.8 ± 0.7 drinks/day, *n* = 5), moderate drinkers (3.8 ± 0.2 drinks/day, *n* = 12), or occasional drinkers (1.2 ± 0.2 drinks/day, *n* = 9). Each woman’s alcohol intake within the clinical research unit was similar to her reported level of consumption for the previous 6–8 years. Sixty percent of the heavy drinkers and 50 percent of the moderate drinkers who consumed more than three drinks per day had significant problems, including delayed ovulation and failure to ovulate (i.e., anovulation). Shortening of the luteal phase also was observed. Menstrual problems did not appear to occur in the women who were occasional drinkers or who were moderate drinkers consuming fewer than two drinks per day. A dose-response relationship appears to exist between alcohol consumption and the frequency of menstrual problems. This notion also is supported by epidemiological surveys showing that prevalence of menstrual disturbances grows with increasing alcohol consumption.

The mechanism of these problems is not entirely clear. However, it is of interest that acute alcohol administration has been reported to raise estrogen levels. Whether this rise is due to an increased estrogen secretion, enhanced conversion of estrogen from precursor substances, decreased metabolism, or a combination of these factors is uncertain. Nonetheless, it has been demonstrated that estrogen administration can disrupt the reproductive cycle, at least in part because estrogen can suppress FSH (Mello et al. 1993). Recall that a rise in FSH early in the follicular phase of the reproductive cycle is crucial to the proper maturation of the dominant ovarian follicle, from which ovulation will occur and the corpus luteum will develop. This knowledge gives rise to the idea that alcohol intake could lead to increased estrogen, inhibiting FSH and disrupting folliculogenesis and subsequent corpus luteum function. In addition, alcohol has been shown to suppress progesterone, the main secretory product of the corpus luteum. Thus, even moderate amounts of alcohol may cause infertility (through suppressing ovulation) and an increased risk for spontaneous abortion (through interfering with the pregnancy-maintaining function of the corpus luteum).

Careful longitudinal studies of the effects of abstinence from alcohol on reproductive abnormalities are not available. Anecdotal evidence, however, indicates that alcohol-induced reproductive abnormalities are reversible upon discontinuing alcohol intake. Not all drinkers have reproductive abnormalities, indicating either variable susceptibility to the effects of alcohol and/or the development of tolerance to its effects. Therefore, some women who consume alcohol will continue to have regular menses and become pregnant.^4^

Women who have ingested alcohol long enough and frequently enough to develop liver cirrhosis are ill and malnourished, putting them in a different category from the otherwise healthy, well-nourished women discussed above. Nonetheless, whether from alcohol or the attendant sickness, women with alcoholic cirrhosis, too, have reproductive and menstrual abnormalities. Their hormonal profiles are somewhat different from those of social drinkers, however. Women with alcoholic cirrhosis tend to have low, rather than high, estrogen and elevated levels of LH and FSH. This pattern is similar to what is normally seen in menopause and suggests that over the long term, alcoholic women may have premature ovarian failure. This area requires further study.

## Prolactin

The hormone prolactin, secreted by the anterior pituitary gland, supports lactation and breast feeding. For most of the other anterior pituitary hormones, the net effect of the hypothalamus is stimulatory; for prolactin, however, the action of the hypothalamus is inhibitory. Much of this inhibitory effect on prolactin synthesis and release is mediated by dopamine, a neurotransmitter delivered from the hypothalamus, although many other hypothalamic regulatory factors may modify prolactin release to a lesser extent. Also in contrast to the other anterior pituitary hormones, which stimulate discrete target organs, no single peripheral tissue clearly feeds back negatively on prolactin release (Molitch 1995).

Human and animal studies on both sexes have demonstrated that both acute and chronic alcohol exposure leads to a stimulation of prolactin release (Emanuele et al. 1993). Alcohol-induced reduction of dopamine’s inhibitory effect may underlie this phenomenon; however, alcohol’s influence on other hypothalamic factors that modify prolactin release also may account for prolactin stimulation. In addition, data from several laboratories show that alcohol applied to the anterior pituitary can stimulate prolactin release, indicating a direct effect of alcohol on the pituitary. Whatever the cause, elevated prolactin frequently is associated with reproductive deficits in both males and females. High prolactin levels can cause impotence in males and disruption of normal ovulatory cycles in females. Clearly, however, elevated prolactin levels are not the only cause for impaired reproductive capabilities: Alcoholic females can have reproductive abnormalities with or without excessive prolactin (Teoh et al. 1995).

The suckling of infants is well known to induce prolactin release; although alcohol normally stimulates an increase in prolactin, the suckling effect is actually diminished (i.e., blunted) in alcohol-ingesting women, impairing breast feeding and leading to negative consequences for infant health (Subramanian 1995).

## Growth Hormone

**Figure 4:**
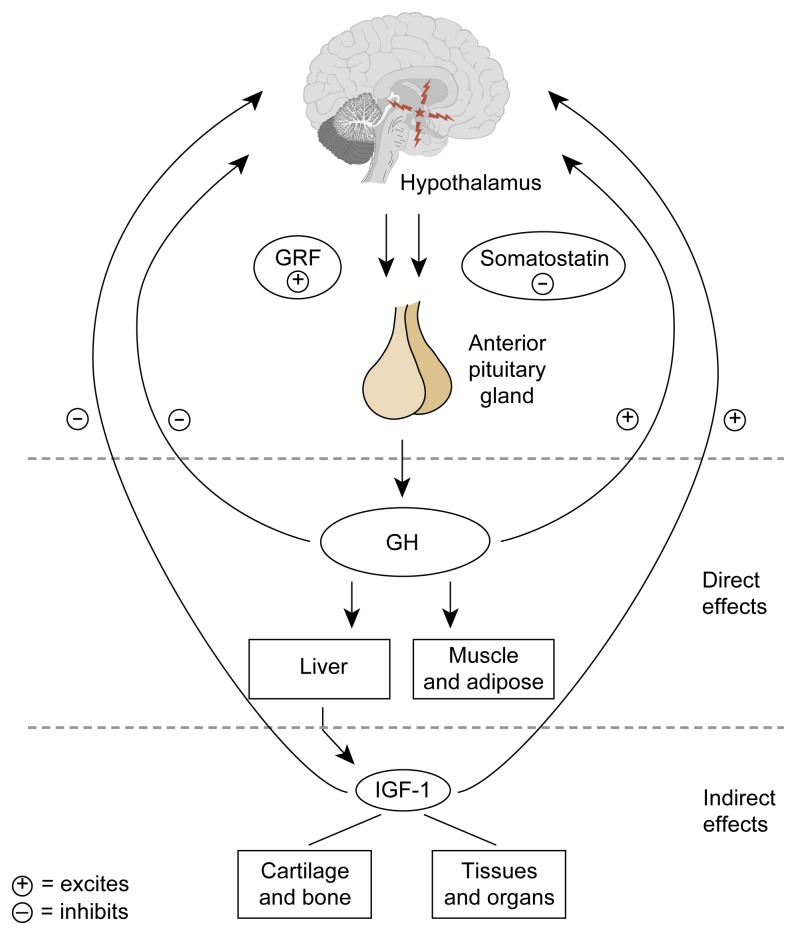
The hypothalamic-pituitary-growth hormone axis. The hypothalamus secretes growth hormone-releasing factor (GRF), prompting the anterior pituitary to secrete growth hormone (GH). The hypothalamus also produces somatostatin, which acts to inhibit GH secretion; the balance of GRF and somatostatin provides the regulatory mechanism for the GH axis. Once GH has been released, a key target for the hormone is the liver, which produces insulinlike growth factor-1 (IGF-1), a chemical that carries out many of the tasks of GH at the cellular level (e.g., normal function of the immune system, carbohydrate and lipid metabolism, and bone growth and development). IGF-1 also feeds back to the hypothalamus and pituitary to reduce GH secretion. GH acts to inhibit its own secretion by inhibiting secretion of GRF and promoting secretion of somatostatin.

The hypothalamic-pituitary-growth hormone (GH) axis functions in a manner similar to the other integrated neuroendocrine systems previously described (figure 4). The arcuate and ventromedial nuclei of the hypothalamus produce large amounts of growth hormone releasing factor (GRF), which is secreted into the hypothalamic-pituitary portal system and then to the anterior pituitary, where it binds to GRF receptors on the pituitary gland. Under GRF stimulation, pituitary GH production is enhanced. Another important hormone in this system is somatostatin. Hypothalamic somatostatin, produced by the paraventricular nucleus, arrives at the pituitary through the portal system, where it activates somatostatin receptors. Somatostatin then inhibits GH secretion. Thus the interplay between hypothalamic GRF and somatostatin tightly regulates the amount of GH being produced by the pituitary gland.

Once pituitary GH has been synthesized and released, a key target for this hormone is the liver, which produces insulinlike growth factor 1 (IGF-1), a chemical that carries out many of the actions of GH (e.g., protein formation and cell growth) at the tissue level. IGF-1 also feeds back at the hypothalamus and the pituitary to reduce GH synthesis and secretion (Molitch 1995). The normal pulsatility of GH release (i.e., its release in pulses) has made it difficult to examine the effects of alcohol on this system. For this reason, IGF-1 has sometimes been used as a marker of integrated GH action and is a more convenient tool for monitoring alcohol-induced changes in the hypothalamic-pituitary-GH axis.

Although an obvious need for normal GH exists during pubertal years, recent studies have demonstrated a beneficial effect of GH on aging tissues (Rudman et al. 1990). The numerous effects of IGF-1 on carbohydrate and lipid metabolism, the cardiovascular system, bone growth and development, and immune-system function may make this hormone extremely important to normal bodily functions, not just in adolescents but also in adults.

Both acute and chronic alcohol exposure consistently have been shown to diminish serum GH and IGF-1 levels in animals and humans of both sexes. Animal studies also have demonstrated a small but significant fall in mRNA for GH in normal adult male rats acutely exposed to alcohol.

In addition to attenuation of basal GH and IGF levels, blunted GH responses to other factors known to increase GH release in healthy humans, such as clonidine, an antihypertensive medication, and insulin-induced hypoglycemia, have been reported after acute alcohol exposure. Both clonidine and hypoglycemia act on the hypothalamus to increase GRF release. It is interesting that acute alcohol given to male rats resulted in a sharp fall in serum GH and IGF levels, yet a concomitant rise in hypothalamic GRF mRNA was noted (Tentler et al. 1993). Because GH is known to attenuate GRF mRNA, the rise in GRF mRNA following alcohol administration is believed to represent an indirect effect secondary to alcohol’s inhibition of pituitary GH (Tentler et al. 1993). Further support for this contention is provided by animal data demonstrating that alcohol directly inhibits pituitary GH secretion but has no direct effect on GRF mRNA in cultured hypothalami.

Thus, the consistently noted profound blunting of GH and IGF-1 levels in both rodents and humans appears to be partially mediated by a suppressive effect at the pituitary level. This lowering of GH and IGF-1 is particularly deleterious to adolescents, who are dependent on these hormones for the normal pubertal process to occur. Older people, however, also suffer negative consequences from a loss of these hormones, such as impaired immune function and muscle weakness.

## Hypothalamic-Pituitary-Thyroid Axis

The hypothalamic-pituitary-thyroid (HPT) axis is of paramount importance to the mammalian organism. Because the metabolic processes of every cell in the body depend on normal amounts of thyroid hormone, disruption of this unit has widespread negative effects on tissue function.

**Figure 5:**
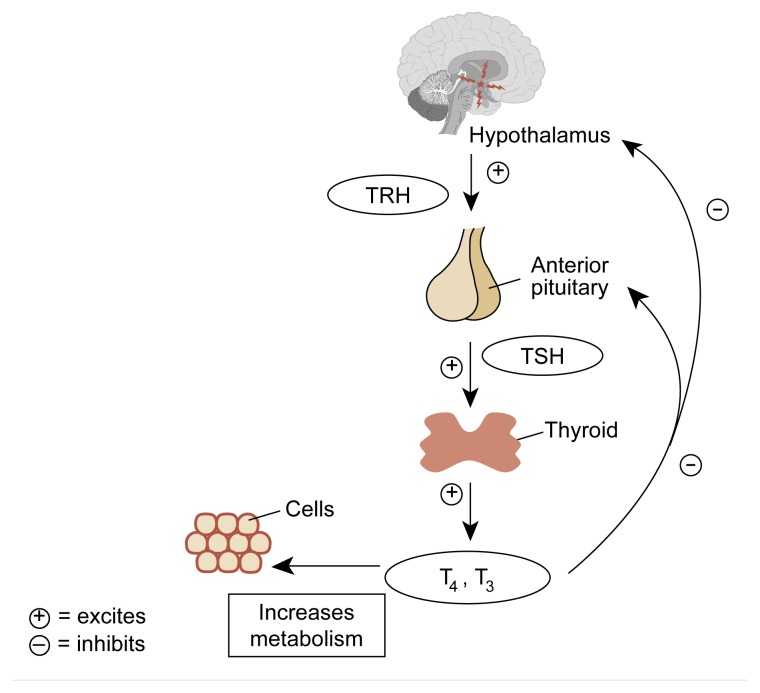
The hypothalamic-pituitary-thyroid axis. The hypothalamus produces and secretes thyrotropin-releasing hormone (TRH), stimulating cells of the anterior pituitary to produce thyroid-stimulating hormone (TSH). TSH then stimulates secretion of thyroxine (T_4_) and triiodothyronine (T_3_) by the thyroid gland, the primary effect of which is to regulate cell metabolism. T_4_ and T_3_ both feed back to the hypothalamus and the pituitary to reduce TRH and TSH secretion. SOURCE: Guyton, A.C. *Human Physiology and Mechanisms of Disease*. 5th ed. Philadephia: W.B. Saunders, 1992. pp. 566–568.

The paraventricular and periventricular nuclei of the hypothalamus produce and secrete thyrotropin-releasing hormone (TRH) into the hypothalamic-pituitary portal system (figure 5). TRH stimulates the thyrotrope cells of the anterior pituitary gland to produce thyroid-stimulating hormone (TSH), which then stimulates the synthesis and secretion of thyroid hormones. The principal thyroid hormones are thyroxine (T_4_) and triiodothyronine (T_3_). Although the thyroid gland secretes both of these hormones following TSH stimulation, most (about 70 percent) of the circulating T_3_ is derived from the liver’s conversion of T_4_. The circulating thyroid hormones, primarily T_3_, feed back in a negative fashion at both the hypothalamic and the anterior pituitary level (Molitch 1995; Utiger 1995). (For reviews of alcohol’s effects on the HPT axis, see Garbutt et al. 1995; Mason et al. 1995.)

Alcohol administered to healthy nonalcoholics does not appear to cause any clinically important change in the two circulating thyroid hormones, T_4_ and T_3_. Furthermore, neither basal nor TRH stimulation of TSH are affected by alcohol. The situation is somewhat different and quite intriguing in alcoholics. Studied during alcohol withdrawal, minor changes, at most, occur in T_4_ and T_3_. Although basal TSH levels are unchanged from levels in control subjects, a blunted TSH response to TRH has been consistently reported during alcohol withdrawal. TRH is an important stimulator of prolactin, and it is notable that the prolactin response to TRH also is blunted during alcohol withdrawal. Since dopamine is a potent inhibitor of prolactin and, to a lesser degree, of TSH, a reasonable hypothesis is that the blunted TSH and prolactin responses were the result of an alcohol-induced excess of dopaminergic activity at the pituitary level. In fact, administration of the dopamine-receptor blocker metoclo-pramide partially restored TSH and prolactin responses during alcohol withdrawal, indicating that part of the reason for the blunted response was too much dopamine or an increased responsiveness to dopamine of the prolactin-producing cells (Garbutt et al. 1995; Mason et al. 1995).

When abstinent alcoholics were studied (Garbutt et al. 1995), baseline T_3_ and, sometimes, T_4_ levels were lower than in nonalcoholic control subjects. The decline in thyroid hormones might not result from alcohol consumption itself but rather from an attendant illness. The fall in T_3_ and T_4_ is a common adaptation of thyroid hormone economy in acute or chronic nonthyroidal illness, a situation known as the euthyroid sick syndrome. (For example, the euthyroid sick syndrome occurs in situations such as sepsis, burns, and major trauma.) In any case, the TSH response to TRH remains blunted in abstinent alcoholics, as during alcohol withdrawal, but the prolactin response normalizes. The reason for this effect is not clear. It does not seem to be caused by excessive dopaminergic inhibition, as it is during alcohol withdrawal; if this were the case, the prolactin response would be impaired as well. Psychologically depressed individuals also have blunted TSH responses to TRH, but associated depression is not the mechanism here, because no relationship exists between personal or family history of depression and blunted TSH responsiveness in alcoholics. Whatever the reason, it is of great interest that young nonalcoholic men with alcoholic fathers (who are at high risk for alcoholism themselves) also tend to have abnormal TSH responsiveness to TRH, usually a blunted response. This finding suggests that TSH change might be a marker for vulnerability to alcohol and might point to some neurobiological abnormality that predisposes people to that disease.

Soon after TRH was isolated and sequenced, it was found not only in the hypothalamus but also widely distributed in the extrahypothalamic central nervous system (Mason et al. 1995). Such mapping studies suggested that the TRH functioned not only as a hypothalamic hormone, with traditional neuroendocrine effects, but also probably as a neurotransmitter that could have a variety of functions, including effects on behavior. One of the earliest behavioral effects observed in animals was TRH’s ability to counteract the hypnotic and motor-impairing effects of alcohol. Along these lines, an exciting effect currently under study is the ability of TRH to decrease craving for alcohol. Experiments have used TA-0910, a degradation-resistant compound (i.e., one that is difficult to destroy) whose chemical structure resembles TRH (i.e., is a TRH analog) (Mason et al. 1995). TA-0910 dose-dependently reduced rats’ consumption of alcohol and increased their intake of tap water during the 24-hour postinjection interval. These early data suggest the possibility that alcohol intake is based in part on deficient extrahypothalamic TRH neural systems and offer the exciting prospect of the use of TRH analogs clinically in the treatment of alcoholism.

## Alcohol, Calcium Balance, and Bone

To form bone that is structurally sound, normal calcium and phosphorous balance is essential. Active vitamin D (i.e., cholecalciferol) is formed primarily in the skin in the presence of sunlight; smaller amounts are obtained through diet. The active form of vitamin D next is metabolized to a more active form in the liver (i.e., 25-hydroxycholecalciferol), then to an even more potent form in the kidney, (i.e., 1,25-dihydroxycholecalciferol) under the control of parathyroid hormone (PTH). The most active form of vitamin D, 1,25-dihydroxycholecalciferol, is responsible for normal calcium absorption from the intestinal tract. The impact of alcohol on calcium metabolism has been explored, and studies generally have confirmed that alcohol will impair calcium absorption from the upper part of the gastrointestinal (GI) tract (i.e., the jejunum and ileum) (Kelly et al. 1990). This impairment in calcium absorption leads to a fall in serum calcium levels, which feeds back to the parathyroid glands, resulting in an increase in secretion by this gland of parathyroid hormone (PTH). Elevated PTH, in turn, leads to calcium resorption or calcium withdrawal from bone, leading to demineralization of the bone and bone disease (i.e., osteoporosis). (For more information on alcohol’s effects on the GI tract, see the article by Bode and Bode, pp. 76–83.) Another school of thought suggests that the most important deleterious effect of alcohol on bone is not mediated through PTH but rather by direct inhibition of the function of bone-forming cells called osteoblasts (Kimble 1997; Klein 1997; Sampson 1997).

Contributing to the problem of bone demineralization is alcohol-induced testosterone suppression. The importance of androgens for maintaining bone mass in adult males is well established (Kelly et al. 1990). Androgens also appear to increase bone formation at critical areas in the long bones of the body and at areas of the bones (i.e., cortical) that promote normal bone growth. Several lines of evidence firmly correlate reduced skeletal mass with reduced testosterone levels in men (Orwoll and Klein 1995): Men who experience testosterone deprivation for any reason throughout their life have decreased bone mass. Thus, low testosterone levels resulting from alcohol ingestion can result in osteoporosis, causing increased risk of bone fractures. A reduction in bone mass of 50 percent has been reported in chronic alcoholics (Feitelberg et al. 1987; Moniz 1994), confirming the magnitude of this problem. Thus, the problem with accelerated osteoporosis in males exposed to alcohol seems to be related to calcium malabsorption with subsequent PTH elevation and low testosterone levels.

Coupled with the problem of osteoporosis is the fact that the increase in PTH release not only encourages more avid calcium absorption from the GI tract but also results in enhanced phosphorous excretion into the urine, with the resultant effect of low serum phosphorous levels (i.e., hypophosphatemia). Hypophosphatemia has profound effects on muscles, resulting in weakness of the shoulder and pelvic girdle muscles, making it difficult for patients to perform simple maneuvers such as rising from a sitting position and climbing stairs. (For more information on hypophosphatemia, see the article by Epstein, pp. 84–92).

## Pancreatic Function

Control of blood glucose is a finely tuned process involving the key hormone insulin, which is produced by special cells of the pancreas called β cells. (For more information on the effects of alcohol on the pancreas, see the article by Apte and colleagues, pp. 13–20.) Insulin synthesis and secretion are controlled by a complex cascade of hormonal signals and enzymes that operate within each β cell, resulting in the release of insulin into the blood circulation. Once insulin reaches its appropriate target cells, especially liver, muscle, and bone, it binds to a receptor and activates a process by which proteins called glucose transporters are produced and brought to the surfaces of the various target cells. They then facilitate entry of glucose into these cells, which can be used immediately for energy or stored for the cells’ future use.

The effect of alcohol ingestion on blood glucose in diabetic individuals is highly dependent on whether the alcohol intake is acute or chronic and whether it is consumed in the fed or fasted state.^5^ Research shows that when nonfasted diabetics drink alcohol acutely, no seriously harmful effect on blood glucose occurs. (For a review of alcohol’s effects on diabetes, see Swade and Emanuele in press.) The effect of chronic alcohol intake is quite different, however. Ben and colleagues (1991) studied 46 diabetics who were “habitual drinkers”; 35 diabetics with no substantial drinking history; and 40 non-diabetic, nondrinking control subjects. One of the parameters examined was hemoglobin A1C values. Hemoglobin A1C is a form of hemoglobin that has a glucose component attached to it (i.e., is glycosylated). The higher the average blood glucose, the greater the percentage of hemoglobin that is glycosylated. Hemoglobin A1C levels reflect average blood glucose control over the preceding 2 to 3 months; higher A1C values indicate poorer blood-glucose control. Hemoglobin A1C values were significantly higher in the diabetics who were habitual drinkers than in nondrinking diabetics; the nondrinking diabetics had hemoglobin A1C values significantly higher than the nondrinking, nondiabetic control subjects.

Ben and colleagues (1991) also measured levels of C peptide, a byproduct of insulin synthesis that is a good measure of insulin secretion. Compared with the nondiabetic, non-drinking control subjects, C-peptide levels were significantly lower in the two diabetic groups. Comparisons of the two diabetic groups, however, showed no significant difference in C peptides. Thus, although the alcohol-drinking diabetics had higher-than-average blood glucose levels (as reflected in the hemoglobin A1C levels) compared with the non-alcohol-drinking diabetics, the two groups’ insulin secretion was equivalent. Thus, the mechanism of elevated glucose is likely explained by an alcohol-associated increase in insulin resistance or the inability of insulin to work properly at the cellular level. This condition of insulin resistance is aggravated by the sedentary lifestyle and poor eating habits of some alcoholics.

In the fed state, chronic alcohol consumption raises blood glucose; in the fasted state, alcohol can produce low blood glucose (i.e., hypoglycemia) in both diabetics and nondiabetics. In addition to simply not eating, alcoholic hypoglycemia occurs for two main reasons. In the fasting state, the body has two major defense mechanisms to prevent hypoglycemia: glycogenolysis and gluconeogenesis. Glycogen, a chemical made up of glucose molecules linked together, is stored primarily in the liver. Glycogenolysis is the process by which glycogen is broken down into its constituent glucose molecules and secreted into the circulation; it is the body’s first line of defense against fasting-state hypoglycemia. Glycogen reserves, however, last only for several hours; when depleted, the body turns to gluconeogenesis, its second line of defense. Gluconeogenesis is the formation of new glucose from amino acids and other chemical substances, a process occurring primarily in the liver. In conditions of alcoholic hypoglycemia, patients are usually starved, and their glycogen reserve has already been depleted. Furthermore, the metabolism of alcohol shuts down the process of gluconeogenesis; consequently, no glucose can be formed. Because of depleted glycogen reserves and impaired gluconeogenesis, the person becomes hypoglycemic. Some of the most profound hypoglycemia seen clinically is alcoholic hypoglycemia, which can lead to permanent neurological consequences, such as paralysis, seizures, coma, or even death.

## Conclusion

The effects of alcohol on the endocrine system have far-reaching consequences not only for hormone production but also for the function of virtually every organ system. Alcohol-related problems include immune dysfunction as a result of disturbances in cortisol, testosterone, GH, and prolactin; reproductive problems; cardiovascular abnormalities stemming from disrupted glucose and lipid balance; and bone disease, among others. The role of hormones, such as TRH, in the genesis of alcoholism is an exciting area of research; findings in this area may produce innovative treatments for the disorder.

## Footnotes

1 For definitions of this and other technical terms, see the central glossary, pp. 93–96.

2 Feedback is a regulatory mechanism for a system (e.g., the HPA axis) in which some of the output or by-products of the system (e.g., glucocorticoids) returns as input (e.g., to the hypothalamus and the pituitary gland). Negative feedback is what occurs when the returning signal (in this case, glucocorticoids) results in a reduction in system output (here, the diminished release of CRF and ACTH). Conversely, positive feedback leads to an increase in system output.

3 For a review of the effects of both acute and chronic alcohol administration to rats, see Rivier 1996.

4 Alcohol ingestion during pregnancy can lead to a variety of defects in the offspring, collectively known as fetal alcohol syndrome (FAS). For more information on FAS, see *Alcohol Health & Research World* Vol. 18, No. 1, 1994.

5 A fed state is one in which a person has consumed normal quantities of food over the 8 hours preceding measurement of blood components or other physiological factors; a fasted state is one in which a person has consumed no food for at least the 8 hours preceding measurements.
